# How can sleep disorders affect our reaction towards external stressors: a lesson from the COVID-19 outbreak

**DOI:** 10.1007/s10072-023-06938-y

**Published:** 2023-08-16

**Authors:** Gloria Castelletti, Francesco Misirocchi, Alessandro Zilioli, Marcello Luigi Salvatelli, Francesco Rausa, Silvia Pizzarotti, Lucia Zinno, Irene Florindo, Giuseppe Pedrazzi, Liborio Parrino, Carlotta Mutti

**Affiliations:** 1 Sleep Disorders Center, Department of General and Specialized Medicine, University Hospital of Parma, Via Gramsci 14, 43126 Parma, Italy; 2 Department of Medicine and Surgery, University of Parma, Parma, Italy; 3 Unit of Neuroscience & Interdepartmental Center of Robust Statistics, Department of Medicine and Surgery, University of Parma, Parma, Italy

**Keywords:** COVID-19, Sleep disorders, Psychological impact, Gender, Dreams

## Abstract

**Background:**

The COVID-19 outbreak produced extensive psychological consequences, especially among vulnerable populations. Sleep was identified as one of the most common “indirect targets” of the pandemia, with up to 74.8% of patients surviving from COVID-19 complaining of new-onset sleep disorders. However, so far, the clinic-psychological impact of the outbreak in patients affected by pre-existing sleep disorders has not been examined in details.

**Materials and methods:**

In the present study, we aim to assess the effect of the COVID-19 outbreak in a cohort of 190 adult patients affected by sleep disorders, compared to 265 age and sex-matched healthy sleepers. The assessment was implemented throughout the use of ad hoc anamnestic questions, exploration of dream content, and validated questionnaires, aiming to capture the broad range of the neuropsychological nuances of the COVID-19 impact.

**Results:**

Subjects with pre-existent sleep disorders faced a more severe impact in terms of sleep quality and amount compared to healthy sleepers, presenting longer sleep latency, reduced sleep efficacy, and greater use of hypnotics and medications. On the other hand, healthy sleepers experienced deeper variation in sleeping habits, sleep duration, and greater impact on dream activity in terms of content, emotionality, and presence of recurrent dreams. Finally, in our sample, being female represents an important aggravating factor in the pandemic experience, both in terms of sleep deterioration and with respect to physical and mental health. For instance, females indeed presented the highest scores of Pittsburgh Sleep Quality Index (PSQI) both in cases and control groups (respectively 10 ± 3.8 vs 7.3 ± 3.9 in cases and 6.6 ± 3.6 vs 6.0 ± 3.4 in controls, *p*-value < 0.001).

**Conclusion:**

Pre-existent sleep disorders and the female sex might represent risk factors increasing the clinic-psychological burden in dramatic scenarios, such as the COVID-19 pandemia, requiring dedicated attention from clinicians.

## Introduction

The COVID-19 pandemia dramatically shocked the life of everyone’s life at a worldwide level. Since its beginning in December 2019, the COVID-19 outbreak yielded to manifold psychological consequences especially among vulnerable populations [1, 2].

Mental issues, including anxiety or depression symptoms, have been detected in a high proportion of patients, representing part of the (frequently hidden) dramatic consequences of this condition [1, 3].

The severity of psychological distress in the COVID-19 era has been associated with financial stressors, work/life balance stressors, younger age, female gender, social isolation, and/or pre-existing health issues [1].

Although the COVID-19-related daily consequences (e.g., anxiety, depressive symptoms) are probably the most noticeable effects, a more subtle impact of the ongoing pandemia persists when the sun goes down, jeopardizing the quality and continuity of sleep of exposed subjects.

Sleep is one of the commonest “indirect target” of the COVID-19 pandemia: according to a recently published meta-analysis, the current global pooled prevalence rate of sleep disturbances is around 35.7% and rising up to 74.8% among COVID-19 survivors [4], with Italy and France presenting the highest prevalence rate (around 55% and 50.8%, respectively) [4, 5].

Sleep has changed during the pandemic era, with a time shift in falling asleep and sleep phases and an overall increase of total sleep time, especially in COVID-affected patients, who commonly refer to an increased odd of troubled sleeping [6]. The pandemic expansion has been accompanied by a concomitant outbreak of the prevalence of sleep disorders, with a specific vulnerability among the COVID-19-infected patients [7].

Changes in sleeping habits have already been described as long-lasting consequences after previous outbreak, such as severe acute respiratory syndrome (SARS), starting in 2002, and Middle East respiratory syndrome (MERS), starting in 2012, which frequently left persistent fatigue, non-restorative sleep, new-onset sleep-breathing disorders, and atypical polysomnographic patterns [8].

The long-lasting consequences of these pathologies in terms of sleep quality probably depend on multifactorial causes, including social isolation, psychological distress, post-traumatic stress disturbances, and, potentially, on the direct effects of the viral infections and/or of hypoxia on sleep-regulatory areas [8].

Papers exploring the complex link between sleep issues and COVID-19 have been often focused on selected samples (mostly cohort of COVID-19-affected patients, healthcare workers, or psychiatric patients), and, so far, to the best of our knowledge, the psychological impact of COVID-19 among patients affected by pre-existent sleep disorders has never been analyzed in details.

Our first aim in this study was to assess the clinic-psychological impact of the pandemia in a large cohort of adult patients affected by different sleep disorders compared to healthy sleepers. The COVID-19 pandemia may be adopted as a model to analyze the reaction of vulnerable populations in extreme catastrophic scenario. We also explored the consequences of this tough period on patients’ dreams content and sleep habits.

## Material and methods

We performed a prospective case-control study at the Sleep Disorders Center, Parma University Hospital, Italy, to assess the impact of the Sars-Cov2 pandemia in adult patients affected by sleep disorders.

Our Sleep Disorders Center covers the entire Parma district area in the North of Italy and a huge part of the Emilia Romagna territory.

Since March 2020, all adult (>18 years old) patients with a diagnosis of sleep disorder were considered eligible for recruitment, while we excluded patients < 18 years old and/or who did not receive a certified diagnosis of sleep disorder after a medical visit performed by sleep experts. All patients with a diagnosis of hypersomnolence disorder and sleep parasomnia performed a whole-night video-PSG with a multi-sleep latency test (MSLT). Patients with sleep-breathing disorders and sleep-related movement disorders were investigated with either nocturnal cardio-respiratory monitoring and/or video-PSG.

A comparable number of healthy sleepers were also enrolled as control population.

Data were collected through phone calls or using an anonymized online survey, respecting patients’ preferences. To ensure replicability of results, all phone calls were performed by the same investigator (GC), a trained psychologist.

At first evaluation (time point 0, t0, coincident in Italy with the first wave of the pandemia), patients were asked to provide information regarding their COVID-19 status (e.g., infected, non-infected, healed from COVID-19), work habits (work schedule, remote working), changes in sleep-wake schedules, ongoing medications, night eating behaviors, and dreams’ frequency and content. Patients were also asked to fulfill standardized questionnaires including Pittsburgh Sleep Quality Index (PSQI) to measure sleep quality [9], 36-Items Short Form Healthy Survey (SF36) to analyze health-related quality of life [10], post-COVID functional status scale to assess subjective limitation on physical functioning [11], and Coronavirus Anxiety Scale (CAS) to screen for dysfunctional anxiety among participants [12].

Finally, all subjects were invited to answer to the same questions and questionnaires 6 months after enrollment, to verify their long-term mental status and sleep habits with respect to the ongoing COVID-19 pandemia (second time point, t1, coincident in Italy with the second wave of the pandemia).

Recruitment and follow-up were conducted between March 2020 and May 2021.

We classified patients in subgroups according to their diagnosis and respecting the International Classification of Sleep Disorders (ICSD).

Therefore, we identified the following subgroups: (1) insomnia (chronic and short-term insomnia), (2) sleep-related breathing disorder (obstructive sleep apnea, central sleep apnea, sleep-related hypoventilation disorders), (3) central disorders of hypersomnolence (inclusive of narcolepsy, idiopathic hypersomnia, and Kleine-Levin syndrome), (4) circadian rhythm sleep-wake disorders (inclusive of delayed sleep-wake phase disorder, advanced sleep-wake disorder, irregular sleep-wake disorder, and shift-work disorder), (5) parasomnias (NREM-related and REM-related parasomnias), (6) sleep-related movement disorders (restless leg syndrome, periodic limb movement disorder, sleep-related bruxism).

**Fig. 1:**
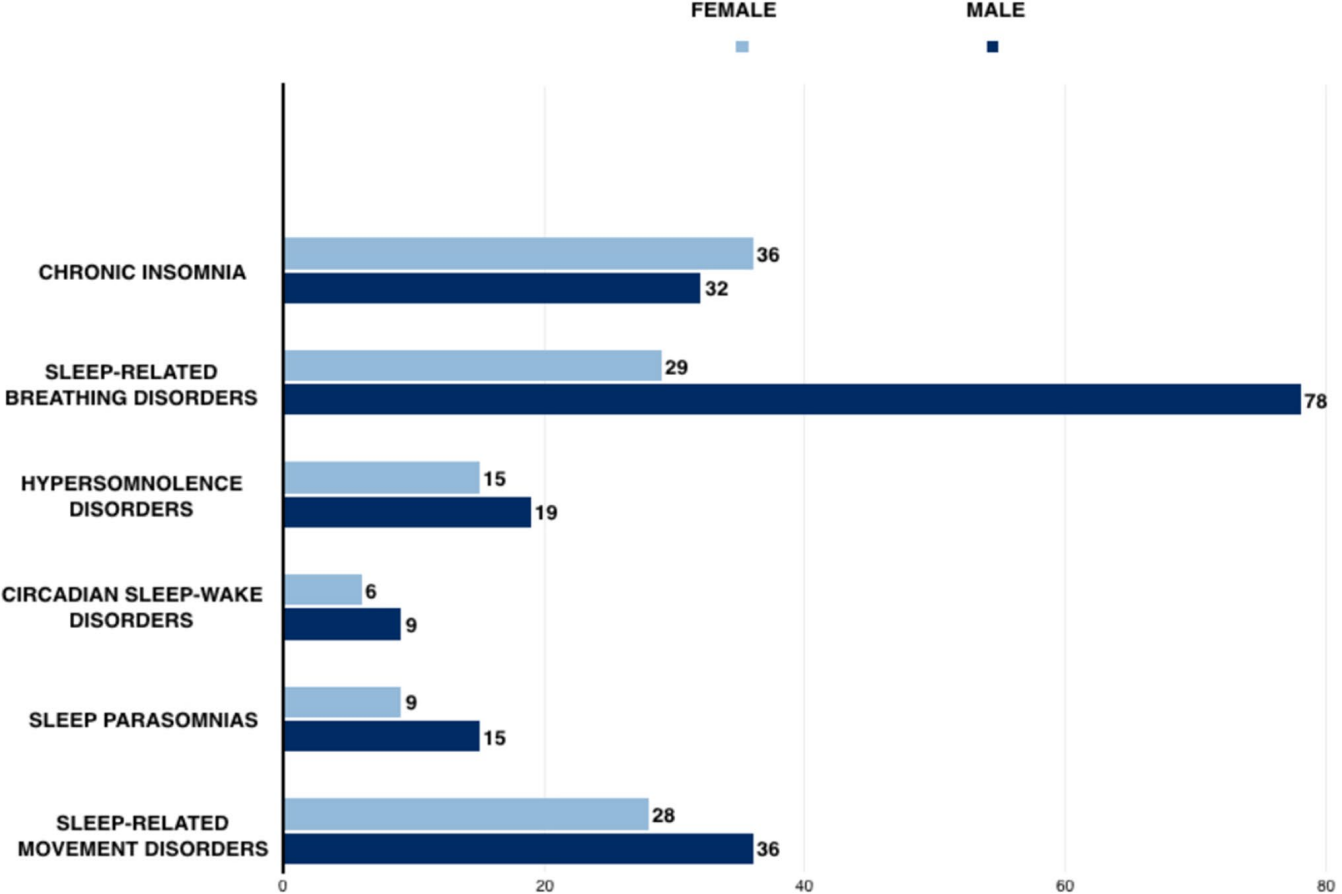
Horizontal histogram displaying patients’ diagnosis, divided per gender

Some patients, belonging to more than one category of sleep disorders, were categorized separately in the subgroup of “polypathological” patients (Fig. 1).

This study was approved by the Local Ethical Committee (reference protocol number: 1434/2020/OSS/AOUPR), and informed consent was requested for all participants.

### Statistical analysis

Statistical analysis was performed using the open-source software Jamovi (v.1.6.7) [13]. All quantitative data were expressed as mean and standard deviation (SD). Qualitative data were reported as absolute frequency and/or percentage. A one-way ANOVA test assessed the differences among the mean values between groups. The normality of the data and homogeneity of the variance were tested by the Shapiro-Wilk test and Levene’s test, respectively. Categorical data were analyzed using Pearson’s chi-square test. Significance was set at < 0.05.

Sample size was determined by power analysis considerations (effect size and accuracy of information), constraints dependent on a rationale of local practical feasibility, and the numerosity of similar studies in the literature.

## Results

Our final sample included 455 subjects, divided into 190 patients (73 females) affected by sleep disorders (“cases”) and 265 (164 females) healthy sleepers (“controls”). Main demographic and clinical characteristics are summarized in Table 1.

**Table 1:** Main demographic and clinic data in cases and controls

	Cases	Controls
No. of subjects	190	265
F/M	73/117	164/101
Mean age (SD)	54.6 (14)	38 (18.2)
COVID-19 infection, *n* (%)	45/190 (23.7%)	37/265 (14%)
Hospitalization due to COVID-19, *n* (%)	19/190 (10%)	2/37 (5.4%)
Work activity, *n* (%)		
- Unemployed	- 47 (24.7%)	- 15 (5.7%)
- Workers	- 143 (75.3%)	- 250 (94.3%)
- Change in working activity	- 16 (8.4%)	- 16 (6%)
- Remote working	- 31 (21.6%)	- 113 (45.2%)
Night eating habit, *n* (%)	30 (15.8%)	47 (17.7%)

Overall, we included 68 patients affected by chronic insomnia (36 females), 107 patients affected by sleep-breathing disorders (29 females), 34 patients affected by hypersomnolence disturbances (15 females), 15 patients affected by circadian sleep-wake disorders (6 females), 24 patients affected by sleep parasomnias (9 females), and 64 patients with sleep-related movement-disorders (28 females). Of these, 93 patients (48.9%) had a single sleep disorder while 97 (51.1%) were categorized as polypathological patients.

### COVID-19 status and clinical need for psychological support

By the end of the study, 23.7% of cases and 14% of controls had been infected by Sars-Cov2, while 10% of cases and 5.4% of controls required hospitalization for COVID-19.

In the years preceding the pandemia, around 7.8% of cases and 8.3% of controls required psychological support for various reasons (including depression, anxiety, panic attacks, eating disorders).

Notably, a significant proportion of cases and controls (22% of cases and 18.5% of controls) required psychological support including psychotherapy, cognitive rehabilitation, or other types of clinical support to deal with new-onset issues related to the pandemia. See Table 2 for further details.

**Table 2:** Psychological status pre- and post-pandemia in cases and controls

	Cases	Controls
Need for psychological support	- Exclusively pre-pandemia 15/190 (7.8%) - Pre and during the first year of the pandemia 31/190 (16.3%) - Only during the pandemia 11/190 (5.7%) - Never 133/190 (70%)	- Exclusively pre-pandemia 22/265 (8.3%) - Pre and during the first year of the pandemia 29/265 (11%) - Only during the pandemia 20/265 (7.5%) - Never 194/265 (73.2%)

### Nocturnal eating behaviors

Notably, a similar number of cases and controls (respectively 15.8% and 17.7%) complained about new-onset nocturnal compulsive eating behaviors during the critical months of the pandemia. Subjects ascribed the night-eating behavior to various reasons including anger, new-onset insomnia, health-related issues, anxiety, or no specific reasons.

### Working habits

With respect to working habits, around 24.7% of cases were unemployed versus only 5.7% of controls.

Around half of the enrolled subjects did not modify their working schedule/duties during the first years of the pandemia. Notably, 45.2% of controls and 21.6% of cases started to work in smart-working regimes.

### Sleeping habits and dreaming mentation

Unexpectedly, variation of the sleeping schedule was more commonly referred to among healthy sleepers compare to patients chronically affected by sleep disorders (respectively 29.5% of cases and 52.8% of controls) and was frequently in the direction of a shorter sleep duration.

Also, a substantial part of controls (39%) noticed changes in terms of dreaming contents and associated emotional reactions, usually with a negative connotation. Variations of the dreams’ content were reported by only 21% of cases, that also frequently presented higher difficulties in remembering the precise dreams’ content.

**Fig. 2:**
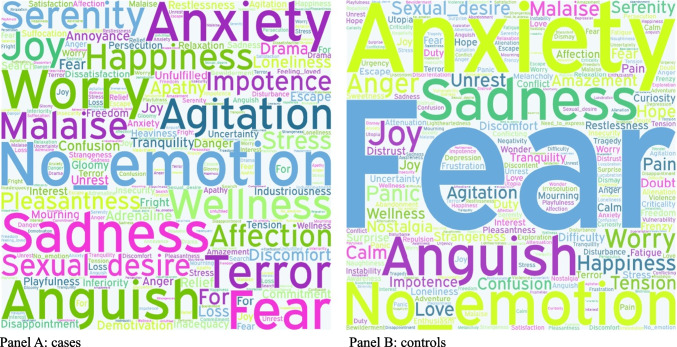
Graphical representation of dream contents in patients (**A**) and controls (**B**). The word cloud of dream content. The size of each word represents the frequency that appears in patients (**A**) and controls (**B**)

Overall, in both cases and controls, the commonest dream-related emotions were fear, anxiety, sorrow, and worry, with some differences between the two groups, as graphically represented in Fig. 2.

### Questionnaire results

#### Sleep quality: PSQI

Mean PSQI scores were above normal values (values > 5 being significant for low sleep quality) in both cases and controls, being, respectively, 8.36 +/− 4.09 and 6.30 +/− 3.54 (see Table 3 for details).

**Table 3:** PSQI subitems in cases and controls

	Cases	Controls
Sleep quality, mean (SD)	1.4 (0.8)	1.3 (0.8)
Sleep latency, mean (SD)	1.3 (1.1)	1.0 (1.0)
Sleep duration, mean (SD)	1.2 (0.9)	0.7 (0.8)
Sleep efficiency, mean (SD)	0.8 (1.1)	0.5 (0.9)
Sleep disturbance, mean (SD)	1.3 (0.6)	1.2 (0.6)
Hypnotics need, mean (SD)	1.2 (1.4)	0.4 (0.9)
Daytime dysfunction, mean (SD)	0.9 (0.9)	1 (0.8)
Tot PSQI result, mean (SD)	**8.4 (4.09)**	**6.3 (3.54)**

In bold mean total values

In details, analyzing PSQI subitems, cases complained about a longer sleep latency (*p*-value 0.002), a shorter sleep duration (*p*-value < 0.001), a lower sleep efficiency (*p*-value 0.005), and a higher need for hypnotic medications (*p*-value < 0.001) compared to controls.

Interestingly, females appear as the most vulnerable category in both cases and controls, presenting the highest scores of PSQI (respectively 10 ± 3.8 female vs 7.3 ± 3.9 male in cases and 6.6 ± 3.6 female vs 6.0 ± 3.4 male in controls, *p*-value < 0.001).

Conversely, no significant differences were detected with respect to subjects’ COVID-19 status: being affected by Sars-Cov2 infection seems not to influence PSQI score (*p*-value 0.680), while some differences were revealed between subjects requiring hospitalization for the disease, presenting a higher, although not significant, score of PSQI compared to non-hospitalized subjects (*p*-value 0.121).

Finally, the change of working habits/duties results in higher PSQI scores in both cases and controls (*p*-value < 0.001, with a mean difference of 2.358 points).

#### Health-related quality of life: Sf36

According to our results, cases presented lower scores at SF36 questionnaire, indicative of lower subjectively perceived health status and health-related quality of life (*p*-value 0.041) (see Table 4). In details, patients affected by sleep disorders complain about higher level of distress in the physical health domains (e.g., pain perception) (*p*-value < 0.001), while controls presented slightly higher vulnerability towards emotional and mental well-being (*p*-value < 0.001).

**Table 4:** SF36 subitems in cases and controls

	Cases	Controls
Physical Component Summary (PCS), mean (SD)	62.2 (23.38)	75 (15.14)
Mental Component Summary (MCS), mean (SD)	62.3 (22.48)	53.64 (23.14)
Physical functioning, mean (SD)	73.2 (25.9)	87.6 (17.5)
Role of limitations due to physical health, mean (SD)	59.7 (41.8)	70.8 (34.8)
Role of limitations due to emotional problems, mean (SD)	64.2 (41.1)	46.4 (43)
Energy level/fatigue, mean (SD)	53.1 (20)	49.1 (20)
Emotional well-being, mean (SD)	61.4 (20.1)	56.1(19.9)
Social functioning, mean (SD)	70.4 (28.9)	62.8 (26.7)
Pain, mean (SD)	63.1 (29.7)	79.5 (22.6)
Tot SF36 result, mean (SD)	**62.2 (20.6)**	**64.3 (15.8)**

In bold mean total values

Once again, gender significantly impacted into questionnaire results, with females presenting lower perceived health quality compared to men (*p*-value < 0.001, mean difference - 6.16 points). Conversely, age did not affect the perceived health quality in neither cases nor controls (*p*-value 0.451).

#### Post-COVID functional status and coronavirus anxiety scale

To assess the clinic-psychological impact of the pandemia, we adopted two validated questionnaires: the Post-COVID-19 Functional Status (PCFS) and the Coronavirus Anxiety Scale (CAS), and we compared results between the first and the second wave of the outbreak (t0 and t1). Results are summarized in Table 5.

**Table 5:** PCFS and CAS during first and second waves of the pandemia in cases and controls

	Cases	Controls
Post-COVID-19 Functional Status (PCFS) - first wave (2020), *n* (%)	0: 45/190 (24%) 1: 36/190 (19%) 2: 35/190 (18%) 3: 49/190 (26%) 4: 25/190 (13%)	0: 86/265 (32%) 1: 80/265 (30%) 2: 49/265 (18%) 3: 45/265 (17%) 4: 5/265 (2%)
Post-COVID-19 Functional Status (PCFS) - second wave (2021), *n* (%)	0: 58/190 (31%) 1: 45/190 (24%) 2: 51/190 (27%) 3: 28/190 (15%) 4: 8/190 (4%)	0: 93/265 (35%) 1: 99/265 (37%) 2: 52/265 (17%) 3: 19/265 (7%) 4: 2/265 (1%)
Coronavirus Anxiety Scale (CAS) - first wave (2020), mean (SD)	3.24 (3.86)	4.36 (4.23)
Coronavirus Anxiety Scale (CAS) - second wave (2021), mean (SD)	2.32 (3.05)	3.04 (3.49)

Cases presented higher scores at PCFS compared to controls, indicating severe perceived physical limitation (*p*-value 0.052). Differently from t0, in the first wave of the pandemia, we did not evidence significant differences with respect to gender, with the second wave of the pandemia females complained higher level of dysfunctional status compared to men (*p*-value < 0.001).

Interestingly, with respect to the anxiety related to the ongoing pandemia, as indicated by CAS results, controls presented slightly higher anxiety scores with respect to cases.

As detailed in Table 4, CAS scores improved in both cases and controls moving from the first to the second wave of the pandemia.

### Questionnaire results according to pre-existent sleep disorders diagnosis

Table 6 summarized mean results of PSQI, SF36, PCFS, and CAS questionnaires in the different groups of patients affected by sleep disorders. Due to the relatively small composition of each subgroup of patients, we did not perform any statistical comparisons between them.

**Table 6:** Questionnaire results according to sleep disorders

Sleep pathologies	PSQI, mean (SD)	SF36, mean (SD)	PCFS1, mean (SD)	PCFS2, mean (SD)	CAS1, mean (SD)	CAS2, mean (SD)
Chronic insomnia	10.7 (3.67)	61.4 (18.5)	1.97 (1.32)	1.44 (1.19)	3.38 (3.89)	2.33 (3.07)
Sleep breathing disorders	7.21 (3.87)	60.9 (21.6)	1.90 (1.47)	1.40 (1.18)	2.76 (3.15)	2.33 (2.87)
Hypersomnolence disorders	8.56 (3.97)	58.1 (21.6)	2.24 (1.35)	1.41 (1.10)	4.03 (3.93)	2.32 (2.63)
Circadian sleep-wake disorders	9.87 (3.87)	52.1 (22.4)	2.60 (1.30)	1.87 (1.41)	5.47 (5.95)	4 (4.23)
Sleep parasomnias	7.79 (3.98)	61.3 (22.3)	2.33 (1.34)	1.38 (1.01)	4.25 (4.48)	2.21 (2.34)
Sleep-related movement disorders	8.11 (4.29)	64.8 (20.3)	2 (1.49)	1.45 (1.25)	3.83 (4.53)	2.56 (3.48)
Polypathological patients	8.86 (4.19)	60.76 (20.67)	2.13 (1.42)	1.47 (1.19)	3.29 (3.77)	2.46 (2.97)

*SD* standard deviation, *PSQI* Pittsburgh Sleep Quality Index, *SF36* Short-form health survey-36, *PCFS1* Post-Covid Functional Status during the first wave of the pandemia, *PCFS2* Post-Covid Functional Status during the second wave of the pandemia, *CAS1* Coronavirus Anxiety Scale during the first wave of the pandemia, *CAS2* Coronavirus Anxiety Scale during the second wave of the pandemia

Notably, higher scores of PSQI were reported in the subgroups of patients affected by chronic insomnia disorder, circadian sleep-wake disorder (CSWD), and hypersomnolence disorder.

Patients affected by CSWD and hypersomnolence disorders presented the lowest values at SF36 test.

At PCFS and CAS, patients affected by CSWD, hypersomnolence disorders, and sleep parasomnias presented the highest scores (indicative of major impact due to COVID-19). Overall, questionnaire results ameliorate moving from t0 to t1 in all groups.

Finally, patients categorized as poly-pathological did not present any significant differences in terms of questionnaire results, except for the PCFS during the first wave of the pandemia, which reveals a significant disadvantage (*p*-value 0.016) compared to healthy sleepers.

## Discussion

To the best of our knowledge, this is the first study exploring the clinical-psychological impact of a catastrophic event such as the COVID-19 outbreak in a large cohort of patients affected by different sleep disorders.

In our sample, in the framework of a tangled relationship between psychological distress and sleep disturbances, being affected by pre-existent sleep pathologies increases the burden of the distress associated with the ongoing outbreak.

According to our results, cases with chronic insomnia disorder, CSWD, and hypersomnolence disorder presented the highest vulnerability with respect to sleep quality, health-related quality of life, anxiety levels, and perceived limitations related to the COVID-19 outbreak.

CSWD patients may have been tested by the novel rhythms imposed by the social isolation and the quarantine, which may have favored, at least in some cases, the delay or advance of circadian rhythm, even among well-treated patients. This higher difficulty in keeping the synchronization with external timekeepers (sport, light, leisure activities) could disrupt biological balance and worsen clinical status in CSWD patients [14].

New-onset insomnia has largely been listed as one of the commonest complications of the psychological distress related to the COVID-19 pandemia, with a larger impact on health-care workers, as confirmed by a recent systematic review [15]. However, so far, the vulnerability of patients affected by chronic insomnia has not been examined in detail. According to our results, a clinical history of chronic insomnia may favor the worsening of quality of life and sleep quality in affected patients.

Although literature data suggest that the pandemia partly represented an opportunity for patients affected by hypersomnolence disorders [16], allowing more time for resting and naps, in our sample, patients affected by narcolepsy and/or other forms of hypersomnolence disturbances experienced high level of anxiety, perceived a reduction in their functional status, and complained a lower sleep quality and quality of life.

Hence, careful attention should be dedicated by the clinicians to patients affected by sleep pathologies during stressful situations.

Besides, our results confirmed the marked vulnerability of females in terms of psychological stress, alterations in mood or cognition, and tendency for hyper-arousal status towards dramatic/stressful scenarios, as already demonstrated by previous studies [17, 18]. This higher frailty of female sex, especially when associated with lower sleep quality, has also been associated with a greater risk of developing post-traumatic stress disorder (PTSD) and/or depressive disorder [17]. Studies in animals and humans confirmed that sex-related factors are responsible for maladaptive coping behaviors in cases of new-onset adversities [19]. Similarly, it has been proven that genetic predisposition, female gender, and environmental stressors can influence the so-called sleep reactivity, corresponding to how the “sleep system” tends to respond to stress. Higher sleep reactivity, lastly related to the dysregulation in the autonomic nervous system and hypothalamic-pituitary-adrenal axis, is common among females and can promote the development of sleep disturbances in this category [20].

Notably, working habits also significantly impact the psychological well-being, with subjects changing their work schedule/routine and complaining about lower sleep quality. The change of the work organization from those in the workplace to the remote form (aka smart working) played a key role in limiting the spread of the disease during the acute phase of the pandemia. However, it has also been linked to many life and work challenges, which can affect quality of life and well-being in exposed workers [21]. Previous investigations highlighted the role for a “work-life balance” (WLB) disorder and an associated decrease in work satisfaction in people forced to change their work to a remote mode [22]. Our study confirms these observations, with remote workers complaining about lower sleep quality, regardless of their gender and clinical status.

Curiously, sleep duration and sleep schedule during the pandemia changed more in healthy sleepers compared to cases, probably reflecting the more consistent adherence to sleep hygiene norms in patients regularly follow in a Sleep-Disorders Center compared to untutored healthy controls.

Sleep hygiene represents one of the most powerful tools in the insomnia primary care [23].

Healthy sleepers referred to more changes in their usual dream content, with a higher percentage of dream-related negative emotions. The impact of the pandemia with respect to dream contents had been largely investigated in a recent Italian web-based survey over more than 5900 subjects [24]. Accordingly, since the beginning of the Sars-Cov2 outbreak, numerous people worldwide reported recalling more vivid dreams, with a higher proportion of nightmares and negative emotional tone. In this direction, a role for confinement and social isolation with respect to sleep and oneiric activity had already been theorized [25].

Unexpectedly, we did not find a clear-cut difference between covid-infected and non-infected subjects in terms of sleep disturbances worsening. To date, several papers have shown how laboratory-confirmed COVID-19 infections are linked with an increased odd of troubled sleeping, sometimes even suggesting a direct causality between conditions [6]. In contrast, our results did not confirm a direct role for COVID-19 infection in terms of sleep worsening. By speculation, we suppose that, at least in milder cases, the emotional response to the infection could feel a pandemic negative effect attenuation, with a sort of event-connected relief. On the other hand, as well highlighted by literature, the pandemic era is largely permeated by anxiety and depression [26], and COVID-19 infection is just one of the abundant lockdown concerns.

The association between COVID-related emotional status and sleep had already been suggested in various samples [27]. Lower sleep quality may reinforce the perception of negative emotions. The association between sleep quality and psychiatric disturbances is so tight that sleep disorders had been suggested as mediators for suicide risk in patients exposed to dramatic situations, such as the COVID-19 pandemia [28].

Our study presents some limitations: all the information were indirectly collected through phone calls and/or online survey. This methodological choice was necessary to guarantee the respect of the norms of social distance during the pandemia. For the same reasons, we were not able to objectively measure sleep duration in patients and controls using actigraphy and/or other forms of sleep recording, to ensure the respect of social distance. However, we selected worldwide adopted validated questionnaires to explore our clinical outcomes, and all the phone calls were performed by the same person, to ensure replicability. Although our data did not allow a deep definition of the neuro-psychological status of patients and controls, we selected ad hoc validated questionnaires, two of whom have been developed to assess the impact of the COVID pandemia. Furthermore, all the information had been collected by one trained psychologist. Given that our sample was not composed by patients affected by psychiatric disorders, we supposed that this psychological evaluation could be enough to screen the more severe psychological consequences of the pandemia.

The two groups (cases and controls) partially differed in terms of gender distribution, as females were more common in the control group. This point however is a frequent limitation in studies enrolling healthy volunteers, due to the higher cooperation coming from females.

## Conclusions

Our study analyzed the clinic-psychological impact of the outbreak in a large cohort of patients suffering from various sleep disorders. According to our results, patients affected by chronic insomnia, hypersomnolence disorders, and CSWD resulted the most targeted ones. Interestingly, healthy sleepers referred a higher tendency for a shift in sleeping schedule and a more relevant impact into their dream mentation habits, with a high proportion of negative connotation, while patients were probably more protected by their sleep-hygiene education. We confirmed the role of the female gender in increasing the risk for distress towards critical scenarios.

Sleep clinicians should be aware that patients affected by sleep disorders, especially females, likely need a careful reassessment during dramatic or stressful scenarios, as their well-being and clinical status can significantly worsen.
